# Treating IgA Nephropathy: Looking at the Future Without Forgetting the Past

**DOI:** 10.3390/jcm14124045

**Published:** 2025-06-07

**Authors:** Rosanna Coppo

**Affiliations:** Fondazione Ricerca Molinette, 10129 Turin, Italy; copporosanna50@gmail.com

**Keywords:** IgA nephropathy, treatment, corticosteroids, targeted released formulation of budesonide, B cells, complement, endothelin

## Abstract

IgA nephropathy (IgAN) is an inflammatory glomerular disease caused by the production of galactose-deficient IgA1 (Gd-IgA1), which induces the formation of autoantibodies and IgA immune complexes (IgAICs) that are ultimately deposited in the mesangium. This event triggers mesangial cell proliferation, cytokine release and complement activation, and both glomerular and interstitial damage, eventually leading to kidney function decline. Persisting proteinuria is the most relevant marker of disease progression. Systemic corticosteroids (CSs), a powerful anti-inflammatory approach, have shown kidney protective effects in early trials involving patients with IgAN at risk of progression with persistent proteinuria. However, later studies raised concerns regarding severe adverse events associated with high doses of methylprednisolone and questioned the long-term benefits. As a result, the KDIGO 2021 guidelines recommended limiting CS therapy to selected patients who accepted the high risk of adverse events. The treatment landscape shifted when reduced doses of methylprednisolone, combined with Pneumocystis pneumonia prophylaxis, demonstrated similar kidney protection compared to full methylprednisolone doses with fewer adverse events. An innovative approach involves a targeted budesonide formulation acting on Peyer’s patches, the main site of Gd-IgA1 production. This treatment showed benefits comparable to systemic CSs, with valuable limitations of adverse events. Several new drugs targeting key pathogenetic events of IgAN are under investigation, with promising results published in recent months. These new therapies target B cell activation (and subsequent Gd-IgA1 production), the complement cascade triggered by IgAIC deposition and the endothelin system, a key amplifier of kidney damage that contributes to the chronicity of IgAN.

## 1. Introduction

### 1.1. Interest in Halting IgA Nephropathy Progression

IgA nephropathy (IgAN), defined by the presence of glomerular deposits of IgA which are dominant or co-dominant over the other classes of immunoglobulins, is a rare disease but a common glomerulonephritis [1,2]. For decades after its identification, IgAN was considered a benign condition, as IgA immunoglobulins are less pro-inflammatory than IgG [3], and the clinical presentation of these subjects is generally mild, with microscopic hematuria and moderate proteinuria or rapidly vanishing gross hematuria. Over time, interest in diagnosing and managing IgAN has grown, as it became clear that IgAN can be a progressive kidney disease, often recognized only through long-term follow-ups [4,5]. In adults with IgAN, 20–40% of any cohort develop end-stage kidney disease (ESKD) with the need for renal replacement therapy within 10–20 years after diagnosis [6]. Patients who do not progress to ESKD over their life have a loss of annual eGFR in the median of 1.2–3.5 mL/min/year as detected in an international cohort of almost 4000 patients followed since kidney biopsy [7]. In recent registry data from the UK, enrolling incident and prevalent patients with proteinuria > 0.5 g/day or eGFR < 60 mL/min per 1.73 m^2^, a progression to ESKD was found in 50% of adults and 20% of children [8]. Almost all patients were at risk of progression to kidney failure within their expected lifetime if eGFR loss was >1 mL/min/1.73 m^2^. Children are also at risk of progression due to their life expectancy even if their post-biopsy eGFR profiles show a trajectory with initial improvement 1–2 years after biopsy, followed by a stabilization and a subsequent decline in eGFR, like what was observed in adults [9]. Moreover, IgAN can recur in grafted kidney, leading to transplanted kidney failure and return to dialysis [10]. Therefore, a strategy is needed to attempt to halt or further slow the relentless progression of IgAN.

### 1.2. Pathogenesis of IgAN: The Initiating Events and the Progression

**Figure 1:**
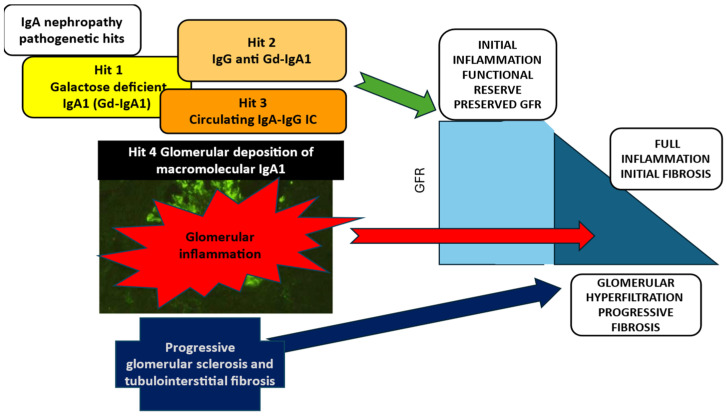
Pathogenesis of IgA nephropathy: the initiating events and the progression.

IgAN is an inflammatory glomerular disease, in which four pathogenetic hits can be identified (Figure 1) [11].

The initiating event is the production of galactose-deficient IgA1 (Gd-IgA1) (hit 1), which induces the formation of auto-antibodies anti-Gd-IgA1 (hit 2), resulting in circulating IgA immune complexes (IgAICs) (hit 3). The glomerular inflammatory reaction induced by the deposition of IgAIC triggers mesangial cell proliferation, the release of cytokines and activation of the complement system, leading to the full development of IgAN lesions (hit 4). In this phase the kidney functional reserve is activated and compensates for the early loss of glomerular function, but the chronic process activates glomerular sclerosis and tubule-interstitial damage, with glomerular hyperfiltration and the chronic kidney disease terminal common pathways ending in the progressive loss of kidney function. Persisting proteinuria and interstitial damage drive the chronic irreversible disease progression [12]. Hence, in the natural history of IgAN, the initiating phlogistic event is shortly followed by the activation of the pro-sclerotic mechanisms, resulting in a mixed unique process that includes active damage as well as consequences of chronic damage, in a balance that changes over time toward the final prevalence of irreversible lesions.

### 1.3. An Overview of the Evolving Recommendations for Treating IgAN

The global vision of the recommended treatment for IgAN has undergone radical changes in recent decades, starting from enthusiastic results of some early RCTs with glucocorticosteroids (CSs) [13,14,15], followed by an unexpected braking from studies showing CS limited long-term benefits and/or safety concerns when using CS high doses or in association with other immunosuppressive drugs [16,17]. This led to the severe restriction of CS use as the first approach to treatment of IgAN, which was recommended to be mostly focused on optimized supportive care (SOC) with renin-angiotensin inhibition (RASB) and lifestyle modifications [18]. This scenario was changed again by the new data on reduced CS dose [19] and the results of a new steroid acting on the intestinal mucosa immune system [20,21]. Meanwhile, new drugs targeting specific pathogenetic mechanisms operating at the initial phlogistic phase as well as during chronicity are being considered, and the field is indeed at an evolving point with a complex road ahead.

## 2. Treatment of IgAN with Corticosteroids (CSs)

### 2.1. Broad-Acting Systemic Glucocorticosteroids

Glucocorticosteroids have been the milestone of treatment of IgAN for decades. In the 1980s three RCTs showed clear evidence of benefits of CS treatment in reducing proteinuria and progression with a loss of renal function [13,14,15]. The results were obtained with 6-month CS regimens, using either a course of three i.v. pulses of methylprednisolone (1 g) on month 1, 3, and 5 followed by oral prednisone (0.5 mg/kg) on alternate days, for six months [13] or a regimen of oral prednisone (0.8–1 mg/kg/day) for 2 months, weaning over 6 months [14,15]. Both protocols induced a reduction in disease progression and proteinuria without serious side effects. A long-term legacy effect was observed for the i.v. protocol [22]. These RCTs were performed without complete RASB since this treatment was not routinely used. Based on these studies, the 2012 Kidney Disease Improving Global Outcome (KDIGO) clinical guidelines [23] suggested to give CS to patients with IgAN and good kidney function (GFR > 50 mL/min/1.73 m^2^) maintaining persistent proteinuria > 1 g/day, despite 3–6 months of SOC with RASB.

Two RCTs performed in the last decade took care of SOC, the STOP-IgAN [16], and the Therapeutic Evaluation of Steroids in IgA Nephropathy (TESTING) study [17]. In the STOP-IgAN trial two corticosteroid-immunosuppressive (CS/IS) regimens were adopted for 3 years (in case of eGFR > 60 mL/min: methylprednisolone pulse protocol, in case of eGFR < 60 mL/min oral prednisone and cyclophosphamide/azathioprine), and full SOC was adopted for treated and placebo subjects, with rigorous RASB and lifestyle changes. Patients in the methylprednisolone pulse arm more frequently than in SOC reached complete proteinuria remission (<0.3 g/day) but without effects on eGFR decline. The safety surveillance reported a high frequency of adverse events in both CS/IS and SOC (36% of patients) arms. A few severe adverse events (SAEs)—obesity and impaired glucose tolerance—were significantly more frequent in the treatment group. The frequency of infections was similar in the CS/IS and SOC groups, but there was one death due to Pneumocystis sepsis in the treatment group. Notably, SAEs were more frequent in patients with reduced eGFR and treated with CS associated with immunosuppressive drugs. In a follow-up observation ten years later, no protection from a 40% decline in eGFR or ESKD was reported [24]. In the TESTING trial, full doses of oral CS were adopted (0.6–0.8 mg/kg/day of methylprednisolone at reducing doses over 6–9 months) versus RASB [17]. The RCT was discontinued during recruitment because of excessive serious adverse events (mostly infections, including two deaths) in patients treated with CS.

Based on these RCTs, the KDIGO 2021 guidelines concluded on the uncertainty over the safety and efficacy of CS treatment for IgAN and suggested focusing mostly on strenuous SOC including a low-sodium diet and maximally tolerated doses of RASB for 3–6 months before discussing risk and benefits of CS with patients who remain at risk for progression with persistent proteinuria > 1 g/day despite SOC [25].

Shortly after the publication of KDIGO 2021, the breakthrough publication of the final TESTING RCT [19] changed again the perspective of systemic CS treatment. In this study, the methylprednisolone dose was reduced from 0.6–0.8 mg/kg/day for 2 months, tapering over 6–8 months to 0.4 mg/kg/day for 2 months, and the prophylaxis for Pneumocystis Jirovecii was added for 3 months. The study involved 257 CS-treated patients (140 with full doses and 117 with low doses) and 246 patients on SOC with careful RASB use. After 4.6 years, a significant effect on the primary end point (40% reduction in GFR or ESKD) was observed in 28.8% of patients in the CS group compared with 43.1% in the SOC group (*p* < 0.001), and low doses were found to be as effective as full doses. SAEs were reported in 10.9% of patients treated with CS and in 2.8% of those in SOC. Patients on reduced doses had SAEs less frequently, but severe infections were reported in 3 cases anyway, although at a lower frequency than in the full-dose protocol, which reported infections in 17 cases (3 fatal). Hence the enthusiasm for the efficacy of methylprednisolone treatment in IgAN with persistent proteinuria despite careful RASB was tempered by the warning of side effects, particularly infections. It is to be mentioned that no attention has been regularly paid to CS toxicity prevention, including a single morning dose or an alternate-day schedule and prescribing a hypocaloric and low-sodium diet, vitamin D supplements and physical activity [26].

The data reported by the recent literature support clear evidence that systemic CSs, which have multifaceted anti-inflammatory activity, are effective in reducing proteinuria and protecting patients with IgAN from kidney function decline at least over the first years. Therefore, systemic CS can be used for the initial treatment of patients at risk of progression, after the careful exclusion of contraindications, avoiding high and prolonged doses and applying lifestyle controls and infection prophylaxis when needed. The remaining risk of adverse events should be balanced with the potential benefits.

### 2.2. A New Horizon for Corticosteroid Treatment in IgAN: Intestinal Immunity Targeted Formulation of Budesonide

The recent research has provided an innovative approach to CS therapy in IgAN, based on a formulation of budesonide (Nefecon) designed to target the gut-associated lymphoid tissue at the Peyer’s patches, a major site of production of Gd-IgA1 located most abundantly at the distal jejunal site [27,28]. Budesonide is a powerful CS with a formula that favors high local activity and has a high hepatic clearance, theoretically limiting systemic exposure [26,29].

The NefIgArd RCT [20,21] investigated 364 patients with IgAN and proteinuria > 0.8 g/g or >1 g/24 h randomized to the targeted release formulation of budesonide Nefecon, 16 mg for 9 months, or SOC. SOC was continued in every patient over 2 years. After 9 months of the Nefecon RCT, a 27% reduction in proteinuria (urinary protein-to-creatinine ratio, UPCR) was observed (*p* < 0.0003 versus placebo), and eGFR was more preserved (difference versus placebo, 3.87 mL/min/1.73 m^2^, *p* = 0.0014). The eGFR benefit observed at 9 months was maintained for 15 months without treatment. Time-weighted average eGFR loss was −2.5 mL/min/1.73 m^2^/year in Nefecon-treated patients versus −7.5 mL/min/1.73 m^2^/year in those in SOC (*p* < 0.0001) with a gain in the total slope of 2.9 mL/min/1.73 m^2^/year (*p* < 0.0001) in favor of Nefecon. The effects on eGFR decline were independent of the baseline UPCR. A median 30% reduction in the UPCR was detected after 9 months of treatment, when SOC only was given, with a further reduction at 12 months, of 47% compared to placebo. A reduction in the UPCR of around 40% compared to placebo was observed till the end of the second year. At final observation, dipstick was negative for microscopic hematuria in 59% of patients treated with Nefecon compared to 39% of subjects in the placebo group.

During the 9-month treatment, related adverse events were reported in 10% of the Nefecon group versus 5% in SOC; the drug was discontinued in 9% of patients in Nefecon and 2% in SOC. The Nefecon group had hypertension (17%), peripheral edema (12%), muscle spasms (12%), and acne (11%), suggesting a partial systemic exposure to the active drug. SAEs related to infection were reported in 3% of patients treated with Nefecon and in 1% of those with SOC. In 2023 the FDA approved budesonide delayed release capsules to reduce the loss of kidney function in adults with primary IgAN at risk for disease progression.

Some observations can be of interest when considering in parallel the results of the two RCTs, which used an intestinal targeted release formulation of budesonide, Nefecon for 9 months (NefIgArd RCT) or systemic methylprednisone for 6–9 months (TESTING RCT) [30].

Patients’ demographic data differed for ethnicity (76% White in NefIgArd versus 76% Chinese in TESTING); however, the median values at the baseline of proteinuria and GFR were surprisingly very similar (proteinuria: 2.7 ± 1.7 g/24 h and eGFR 56.1 (45–70) mL/min/1.73 m^2^ in 182 patients treated with Nefecon; proteinuria: 2.55 ± 2.45 g/24 and eGFR 56.1 (43–75) mL/min/1.73 m^2^ in 136 patients and then treated with methylprednisolone for 6–9 months). The effect of methylprednisolone was observed after 3–6 months, while Nefecon showed significant effect after 9 months; however, the antiproteinuric effect was similar with 40–50% of proteinuria reduction lasting over 1–2 years. Nefecon and methylprednisone showed in the two RCTs an increase in GFR at 3–6 months and a subsequent GFR decline over 2 years. Patients in Nefecon had a loss in GFR of −6 mL/min/1.73 m^2^, while SOC had −12 mL/min/1.73 m^2^ over 2 years of follow-up. Patients on methylprednisolone had a GFR loss of −2. 5 mL/min/1.73 m^2^/year, while the SOC arm had a loss of −4.9 mL/min/1.73 m^2^/year over 3.5 years in median follow-up.

From a face-to-face analysis of these RCTs, the benefits provided by systemic CS and the targeted-released formulation of budesonide are rather similar, with similar antiproteinuric effects and eGFR decline protection over the first years. The more frequent and severe adverse events observed using systemic CS, also at reduced doses, would suggest adopting an intestinal targeted-released formulation of budesonide; however, a direct comparison between the two treatments is not available, and side effects related to CS systemic exposure were also reported in Nefecon-treated patients. The beneficial effect of Nefecon on the long-term follow-up of patients with IgAN should be further investigated. Notably, the difference in cost prevents its use in many countries.

## 3. New KDIGO to Be Published in 2025

In the years following the publication of KDIGO 2021 guidelines, several studies indicated that the concept of approaching treatment of IgAN in patients with persistent proteinuria > 1 g/day based on RAS inhibition and SOC was not satisfactory. Despite optimized SOC, the long-term follow-up of patients enrolled in the STOP-IgAN RCT showed that 50% of patients progressed to a 40% loss of eGFR or ESKD after 7.4 years [24]. Moreover, in recent RCTs the eGFR loss in patients with optimal SOC was about −5 mL/min/1.73 m^2^, which is considered unacceptable. Recent data from the UK registry for rare disease (RADAR) indicated that the risk of developing kidney failure within a lifetime is high, particularly if eGFR at enrollment is <60 mL/min, or/and proteinuria is >0.5 g/day [8]. Meanwhile it became clear that the proteinuria threshold > 1 g/day was too high to select patients at risk of progression, hence excluding from the active treatment approach those with lower levels. Indeed, from the Validation Study of the Oxford Classification in IgA nephropathy (VALIGA) study, persistent proteinuria between 0.5 and 0.9 g/day was predictive of long-term kidney survival in a large cohort of 1034 patients of IgAN from several European countries [31], and the RADAR study [8] indicated that a lower threshold of proteinuria > 0.4 g/day was a significant risk for progression to kidney failure. Finally, several new drugs targeting individual pathogenetic factors have been reported and/or are on the study pipeline, suggesting a new revolution in treating IgAN.

Based on these data, in 2024 KDIGO prepared new guidelines, which were available for public review and are now about to be published in the final form (KDIGO-IgAN-IgAV-Guideline-Public-Review-Draft-Data-Supplement_August-2024, n.d.). The main changes include the identification of patients at risk on a proteinuria threshold of ≥0.5 g/day in patients while on or off treatment. Moreover, the target of proteinuria reduction is set to values lower than 0.3 g/day, possibly needing multiple drug interventions.

The major news in the next KDIGO guidelines is that the IgAN patients at risk of progression should receive simultaneous treatments with a double-targeting approach: one side is aiming at preventing or reducing the immune complex-mediated glomerular damage, and in parallel the other side is aiming at limiting the consequences of the existing damage on glomerular loss and hyperfiltration. The list of new drugs active on either process is growing, and it is challenging to follow the entry of new treatment options that progress to authority approval and distribution to patients.

The treatment of IgAN should target (a) the initial pathogenetic event of production of Gd-IgA1, (b) the full explosion of inflammation, led by complement activation, (c) the non-inflammatory progression, previously dominated by RAS inhibitors, now challenged by anti-endothelin A drugs (ERA), dual endothelin and angiotensin receptor antagonism (DEARA), sodium–glucose cotransporter-2 inhibitors (SGLT2is) and mineral corticosteroid receptor antagonists (MRAs). Other drugs are on the pipeline, but we will focus on the most relevant recent publications of phase 2 or phase 3 RCTs, which in some cases led to FDA and EMA approval.

## 4. Treatment Targeting the First Pathogenetic Hit of IgAN: The Synthesis of Gd-IgA1

Polymeric IgA detected in kidney deposits of IgAN is the typical product of the mucosal-associated lymphoid tissue (MALT), and the synthesis is elicited by environmental antigens, microbiota and their products [32]. In IgAN, Gd-IgA1 can be produced by gut-associated lymphoid tissue (GALT) as well as by tonsils and nasopharynx-associated lymphoid tissue (NALT). The GALT surface of 300 m^2^ largely covers more than half of the IgA synthesis from other MALT sites (including the limited NALT). Hence, the Peyer’s patches, lymphoid follicles mostly represented in the terminal small intestine, are the most active site of production of Gd-IgA1 in patients with IgAN [27,28,33]. The treatment with the targeted released formulation of budesonide is considered by KDIGO to be the only steroid treatment able to reduce the levels of pathogenetic forms of IgAs (Gd-IgA1) and IgAICs [34].

However, the reduction in Gd-IgA1 can be obtained at an early stage by acting on lymphocytes. In the MALT, the antigens prime naïve B cells through T cell-dependent and T cell-independent mechanisms [35]. The T cell independent pathway is activated via interleukins (lL-6 and IL-10), transforming growth factor (TGF-β), B cell activating factor (BAFF or BLyS), and a proliferation-inducing ligand (APRIL). The BAFF and APRIL bind the TNF receptor transmembrane activator (TACI) and promote B cell differentiation and proliferation. The BAFF and APRIL are tumor necrosis factor (TNF) superfamily cytokines that play a fundamental role in regulating IgA production by B cells and the survival of IgA-producing plasma cells [36]. The BAFF is particularly active on B cell maturation, survival, and transition to mature B cells with immunoglobulin production. APRIL has a more precise role in the later stages of B cell differentiation and survival of long-lived plasma cells, located in the bone marrow and in the MALT, to produce antibodies. The role of the APRIL in IgAN is supported by several observations including IgA class antibody production and Gd-IgA1 synthesis (particularly in patients with IgAN) [37].

In conclusion, anti-APRIL/BAFF drugs as well as Nefecon can reduce the levels of Gd-IgA1. Data on the effects of systemic CS on the levels of Gd-IgA1 are expected.

## 5. Existing Therapies to Inhibit Lymphocyte Proliferation

The first drug used to inhibit B and T lymphocyte proliferation was mycophenolate mofetil (MMF), which had conflicting reports, mostly negative in early small studies in White subjects and largely positive in trials in Asian subjects [38]. A more recent Chinese study [39] enrolled incident patients with biopsy within one month and active disease including proteinuria > 1.0 g/day, eGFR > 30 mL/min, and crescents involving between 10 and 50% of the glomeruli or endocapillary hypercellularity or glomerular necrosis and limited tubular atrophy/interstitial fibrosis. The protocol had two arms: (a) prednisone, 0.8–1 mg/kg /day, for 2 months, tapering in 4 months, and (b) MMF, 1.5 g/day, added to half-dose prednisone (0.4 mg/kg/day). The primary endpoints were complete remission and changes in active proliferative lesions on a repeat biopsy. Results showed that the two regimens had similar benefits with fewer adverse events in patients receiving MMF and a half dose of prednisone. The Mycophenolate Mofetil Among Patients with Progressive IgA nephropathy (MAIN) trial [40] enrolled 170 Chinese patients with IgAN in an open-label randomization to placebo or MMF at an initial dose of 1.5 g/day tapered to 0.75–1 g/day for 6 months. MMF was found to significantly reduce the risk of disease progression with respect to SOC alone. The AEs were limited to mild and tolerable gastrointestinal symptoms. Infections (pneumonia) were reported in 16% of MMF but also in 10% of SOC.

In a real-world setting in the Chinese national database recording data from 3964 patients with IgAN who initiated treatment within 30 days of renal biopsy [41], two groups of 1973 patients on SOC (RASB) or in immunosuppressive care (CS with or without MMF) were selected by propensity score and compared. IS treatment was associated with a 40% lower risk of the primary outcome of 40% decrease in eGFR or ESKD. The results were comparable in CS monotherapy and MMF alone and in patients with various proteinuria and eGFR levels. A great benefit was observed in patients with endocapillary hypercellularity (E1) and segmental glomerulosclerosis (S1) and without severe tubulo-interstitial lesions (T0-1).

These studies had the limitation of being performed in Chinese subjects only; however, large cohorts of accurately followed patients were reported. The different response in Chinese and Caucasian patients to MMF has never been proved by a comparative “ad hoc” planned study. MMF is commonly used in association with CS in active patients in large outstanding Western Centers [42].

## 6. New Treatments Targeting B Cells and Plasma Cells

The first approach to B cell treatment was to test rituximab, a CD20 antibody, in a small RCT enrolling 34 patients with IgAN and persistent proteinuria > 1 g despite RASB [43]. The results were disappointing, failing to show any reduction in proteinuria or kidney function protection. Moreover, CD20 depletion did not modify levels of Gd-IgA1 or anti-Gd-IgA1 antibodies, suggesting that CD20 was not implicated in the initial pathogenetic event. Indeed, the depletion of CD20^+^ B cells by rituximab was reported not to affect gut-resident plasma cells [44].

The B cell target was moved to CD38 B cells and plasma cells [45], considered to be more closely involved in the formation of Gd-IgA1 and the corresponding autoantibody. Felzartamab, a recombinant fully human monoclonal antibody against CD38, is under evaluation in a phase 2a trial (IGNAZ; NCT05065970) (Table 1). The intention to treat analysis was performed in 48 subjects with IgAN enrolled across immune-mediated kidney diseases, including membranous nephropathy, lupus nephritis and antibody-mediated rejection [46]. In different doses (two to nine doses in 15 days–9 months) showed a reduction in UPCR) over 6 months, which persisted ten months after the last dose. Rapid and durable reduction in IgA and Gd-IgA1 was also observed, lasting 10 months after treatment. The AE were of moderate intensity and mostly infusion-related reactions at the first administration.

The pivotal role of BAFF and APRIL in B cell activation, leading to the production of Gd-IgA1, supported the rationale for testing drugs targeting these two TNF superfamily cytokines [35,36,37]. Sibeprenlimab, a humanized IgG2 monoclonal antibody that binds and neutralizes APRIL, was tested in a phase 2 ENVISION trial (NCT04287985) [47]. Different doses of Sibeprenlimab (2, 4, 8 mg/kg i.v.) were administered monthly in 155 patients (117 treated and 38 placebo) to assess efficacy and safety. A significant reduction in proteinuria of −62.0 ± 5.7% was detected with 53% reduction as compared to placebo with the highest drug dose. Proteinuria complete remission was observed in 26.3% of treated patients, and in 2.6% of patients receiving placebo. Levels of Gd-IgA1 were reduced by 40% and IgAICs by 66% after 12 months with Sibeprenlimab 8 mg/kg/iv monthly. Both Gd-IgA1 and IgAIC levels had a rebound 5 months after treatment discontinuation. In an explorative analysis, kidney function was protected as in the 38 patients receiving the dose of 8 mg/kg the treatment difference in the eGFR slope relative to placebo was 5.08 (0.5 to 9.6) ml/min/1.73 m^2^ with an eGFR loss of −1.5 mL/min/1.73 m^2^ in patients receiving the highest doses with respect to placebo, −7 mL/min/1.73 m^2^. Safety was like placebo, and adverse events (78% versus 71% AEs) were mostly mild, without increased risk of infection.

Zigakibart (BION-1301) is a novel anti-APRIL humanized monoclonal antibody tested that provided proteinuria and Gd-IgA1 reduction in phase 1 and 2 studies [48] under evaluation in a phase 2 ongoing study, BEYOND (NCT05852938).

Atacicept, a fully humanized fusion protein acting as a decoy receptor neutralizing both APRIL and BAFF, was used in a phase 2 study, ORIGIN 3 (NCT04716231) [49], in the combined group of 75 mg and 150 mg, a once-weekly s.c. injection resulted in a reduction in the UPCR at 36 weeks by 31%. Circulating Gd-IgA1 levels were reduced by 40%. Safety was like the placebo (AE, 73% in treatment and 79% in the placebo groups). The long-term open-label extension study recently reported the effects of atacicept, 150 mg s.c. weekly in 113 subjects with IgAN for 60 weeks after the end of the phase 2b study [50] and followed up to 96 weeks (31 patients in the former placebo group were treated for 60 weeks). Patients showed a sustained decrease in the UPCR (−52 ± 5%) versus baseline and in Gd-IgA1 (−66 ± 2%) and in the percentage of patients with hematuria (−75%). The eGFR annualized slope over the 96 weeks was −0.6 ± 0.5 mL/min/min/1.73 m^2^, unchanged after the 36 weeks of blind treatment. The drug was well tolerated, discontinued in two cases for mild and transient AEs (injection site pain and Hepatitis B positive test). Serious infections (in three cases) were resolved without sequelae, and no opportunistic or fatal infections occurred.

Similar encouraging data are reported in preliminary reports with telitacicept (NCT05596708) [51] and povetacicept (NCT05732402) [52].

In conclusion, B cell regulation via the inhibition of the APRIL/BAFF is a promising new treatment for patients with IgAN. A significant antiproteinuric effect was found in parallel to Gd-IgA1 reduction. The selection of patients more likely to respond to these drugs and the duration of treatment should be a matter of further investigations.

## 7. New Treatments Targeting the Amplification of Glomerular Inflammation: The Complement Cascade

In IgAN, complement activation plays a pivotal role in the development and perpetuation of inflammation following IgAIC deposition [53]. The absence of C1q suggests that complement is activated via alternative and lectin pathways, and activation occurs after IgAIC deposits within the glomerular area [54]. The attempt to inhibit the lectin pathway by narsoplimab, a fully human monoclonal antibody targeting the lectin regulatory enzyme Mannan Binding Lectin Serine Peptidase MASP2, was interrupted after the finding of no effect on proteinuria at interim analysis of the ARTEMIS phase 3 RTC (NCT0308033) [53].

The inhibition of the alternative pathway seems presently the most appropriate approach. Iptacopan specifically binds to factor B and inhibits the alternative complement pathway. It was tested in the phase 2 APPLAUSE study (NCT04578834) [55] at doses of 19, 50, or 150 mg for 6 months in 112 patients (87 in treatment and 25 in placebo). A dose-dependent proteinuria change was detected at 3 months or 6 months. Mild adverse events were reported and not related to the drug. The interim analysis of 250 patients in the ongoing, phase 3 APPLAUSE-IgAN RCT testing Iptacopan 150 mg for 9 months detected a significant reduction in proteinuria (38.3% lower with iptacopan than with placebo) [56]. The reduction in proteinuria was consistent across subgroups with different geographic regions, baseline proteinuria, GFR, hematuria, MEST-C scores, or co-treatment with SGLT2i. High levels of the membrane attack complex (sC5b-9) found at baseline returned to normal levels. The safety profile was good with no increase in the risk of infection in the treatment group. Iptacopan was approved in 2024 for the reduction in proteinuria in adults with IgAN at the risk of rapid disease progression (UPCR ≥ 1.5 g/g).

The C5 factor is the target of the Cemdisiran small interfering RNA, which reduces hepatic C5 production. In the Phase 2 ALN-CC5 study (NCT03841448) [57], Cemdisiran (600 mg s.c.) was administered every 4 weeks for 36 weeks to 30 patients (9 in placebo). Proteinuria was reduced by −37% with respect to placebo. Moreover 77.3% of Cemdisiran-treated patients showed a reduction in hematuria versus 22.2% of the placebo group. Adverse events were mild or moderate and transient.

A recent report on ravulizumab [58]—a long-acting complement C5 inhibitor targeting the terminal complement pathway—tested in the phase 2 study SANCTUARY RCT (NCT04564339), showed in 66 patients enrolled a significant reduction in proteinuria (30,1% treatment effect versus placebo). Ravulizumab was well tolerated with an adverse event profile like that for placebo. The immediate and complete inhibition of the terminal complement cascade was detected, and C5 concentrations were undetectable.

Complement inhibition, targeting alternative and common complement pathways, has provided interesting data on rapid and relevant proteinuria reduction in patients with IgAN. As for other new drugs, the selection of patients to benefit from this treatment as well as the duration of drug exposure should be a matter of additional investigation.

## 8. The Expanded Supportive Care (Non-Immunologic Treatment) for Patients with IgAN: SGLT2 Inhibitors and Anti-Endothelin A

The therapy targeting the pathogenetic events of IgAN should be started in the early phases, but it must be added on an optimized SOC targeting the chronic and sclerotic progression of the disease (Figure 1). This target remains all over the natural history of IgAN and must be addressed for life.

The introduction of RASB in the treatment of IgAN across the age spectrum was fully recognized since KDIGO 2021 as a pillar of the SOC [25]. However, as mentioned above, several patients remained at risk of CKD progression despite prolonged RASB and optimized SOC; hence, great interest was attracted by new drug primarily tested in diabetic patients, found to provide a reduction in proteinuria and protection from eGFR loss.

The first impressive report showed that SGLT2 inhibitor dapagliflozin reduced the progression of a subgroup of 270 IgAN patients to the endpoint of 50% eGFR loss or ESKD with a favorable safety profile [59]. Proteinuria was reduced by 26% relative to placebo. Dapagliflozin was approved by the US FDA and EMA for CKD in adults, including IgAN.

Sparsentan, a dual endothelin and angiotensin II receptor antagonist (DEARA), provided excellent results in the PROTECT Phase 3 RCT (NCT03762850). Sparsentan (400 mg) or irbesartan (300 mg) was distributed to 404 adults with IgAN, eGFR ≥ 30 mL/min/1.73 m^2^ with persistent proteinuria >1 g/day despite 3 months of optimized SOC. The interim analysis showed a change in the UPCR at week 36 of 49% versus 15% in placebo [60]. In 2023, Sparsentan received FDA accelerated approval for the reduction in proteinuria in IgAN at risk of rapid disease progression (UPCR ≥ 1.5 g/g). In the final report after 2 years [61], proteinuria remained significantly lower in the sparsentan group (reduction of 31% versus 11%). The eGFR 2-year chronic slope, starting from week 6 to week 110, was significantly different (*p* = 0.037). Protection from 40% eGFR reduction, ESKD, or all-cause mortality was significantly better in the sparsentan group. FDA granted full approval to sparsentan in 2025 for slowing kidney function decline in adults with IgAN at risk of disease progression based on final PROTECT trial data.

Selective endothelin A receptor antagonists (ERA) represent a new exciting target. Recent reports provided excellent data on selective endothelin receptor antagonists in addition to RASB, which may allow fine dose tuning and possibly stronger ET-A receptor inhibition than DEARA.

Atrasentan, a selective ET-A receptor inhibitor, was tested in the phase 3 ALIGN RCT (NCT04573478). The interim analysis of 270 patients in week 36 showed a significant reduction in the UPCR in treated patients, 36.1% compared to placebo [62]. Fluid retention was more frequent in the atrasentan group (11.2% versus 8.2%) but did not lead to drug discontinuation. No cases of cardiac failure or severe edema occurred. In 2025, atrasentan received FDA accelerated approval for the reduction in proteinuria in adults with IgAN at risk of rapid disease progression.

The ET-A receptor SC0062 NCT05687890 [63] differs from sparsentan and atrasentan because of a higher selectivity for ET-A compared with ET-B receptors, which may be relevant for both efficacy and the safety profile, particularly fluid retention and edema. In 131 patients with IgAN, the percentage change from the UPCR after 12 weeks of treatment with various doses of SC0062 was significant (up to −38.1% versus placebo), without an increase in peripheral edema.

The new mineralocorticoid receptor antagonist (MRA) finerenone may provide additional benefits when added to RASB, as reported in a recent meta-analysis [64]. The ongoing FIND-CKD RCT (NCT05047263) is investigating patients with CKD due to several diseases, including IgAN [65].

According to the new KDIGO publication, all patients with IgAN should receive optimized SOC, which includes blood pressure control, lifestyle modifications and proteinuria control using traditional RAS inhibitors at the maximally tolerated doses while simultaneously preventing or reducing immune complex-mediated glomerular injury. RAS inhibition is now being implemented by new drugs, including SGLT2i, ERA, or DEARA, which have received or are about to receive full FDA approval for patients with IgAN at risk of progression. The list of available drugs is rapidly evolving and expanding. However, there is currently no direct comparison of benefits and side effects between these treatments. Furthermore, the cost of these new drugs compared to traditional RAS inhibitors makes them inaccessible in some countries.

## 9. Final Considerations

The treatment of IgAN is a rapidly evolving field [66]. New therapeutic options are emerging, and research is now focusing on drugs targeting specific factors acting in the pathogenesis or the progression of IgAN. These studies provide a unique opportunity to identify therapeutic targets that may lead to a precision medicine approach. However, this process will take time, as many ongoing or recently published studies were primarily designed to demonstrate the efficacy of experimental drugs in reducing proteinuria—typically after nine months of treatment—and slowing eGFR decline over two years of continuous treatment or post-treatment observation. The trial designs were largely driven by the need for industries to prove predefined standard benefits and obtain the authority’s approval. This step was due to regulatory requirements; however, for real-world application, it should be noted that patients in these studies were generally selected based solely on persistent proteinuria despite SOC without considering (a) duration of the previous disease, (b) clinical and histological signs of activity and chronicity at the start of treatment, or (c) the presence of specific biomarkers of the treatment target in circulation or in kidney tissue.

For the new drugs, we currently lack data on the duration of treatment required to achieve long-term benefits. The potential adverse effects of long-term exposure are also unknown. Moreover, in the absence of a comparative study, clinicians may be tempted to use all possible medications simultaneously (although each might be appropriate individually), such as combining SGLT2i and anti-endothelin with RAS inhibitors to optimize SOC. Similarly, in active cases, targeting the immune process with the intestinal released formulation of budesonide combined with an anti-complement agent may be tempting option. However, we lack data on the efficacy and safety of such combinations. Small real-world series that may be reported at scientific meetings will provide only limited insights into treatment indications in a disease as complex and multifaceted as IgAN, especially given the expected variability in patient selection criteria. The natural history of IgAN is long and may include phases of activity and remission following treatment. Understanding the most effective treatment combination and optimal timing will require collaborative data analysis on large international cohorts. Artificial intelligence may be a valuable tool to analyze large datasets and derive personalized treatment strategies. Currently, excellent flow charts are available in publications [66,67] and online that can help guide clinical decisions, but each algorithm is likely to be revised in the coming months or years as new evidence emerges.

The high cost of these new drugs is a major concern, making them unavailable for public distribution in most developing countries. Many patients who are not covered by public or private insurance cannot afford these new treatments, leading to potential frustration due to the perceived loss of therapeutic opportunities.

It is important to remember that some “older” treatments for IgAN, such as CS and MMF, still have a role both in countries where patients cannot afford newer drugs and in developed countries. In patients with active IgAN, identified by well-established clinical and histological markers (including rapid kidney function decline, the presence of severe microscopic hematuria and histological indicators) and not based solely on proteinuria, treatment with proven benefits and limited adverse events should be offered, in addition to SOC. Once irreversible progression is established, it cannot be halted. In real-world settings, the side effects of CS pulses are generally limited when administered in selected cases and with appropriate precautions [26,68]. Moreover, studies have reported variable dosing regimens with similarly favorable outcomes [69]. MMF at reduced doses has been shown to be effective and safe [40]. Oral CS at a moderate dose, or methylprednisolone pulses, and MMF remain part of the current therapeutic arsenal for IgAN, guiding us toward the future without neglecting valuable lessons from the past.

## Figures and Tables

**Table 1:** Recent clinical trials in IgAN. Legend: APRIL: a proliferative inducing ligand (APRIL); BAFF: B cell activating factor; MASP: Mannan Binding Lectin Serine Peptidase; C factor B: complement factor B; and C5: complement factor 5.

Treatment Target	Mechanism of Action	Drug	Study	Phase	Identifier
B cells and plasma cells					
	CD38	Feltarzamab	IGNAZ	IIa	NCT05065970
	APRIL	Sibeprenlimab	ENVISION	II	NCT04287985
	APRIL	Sibeprenlimab	VISIONARY	III	NCT05248646
	APRIL	Zigakibart	BEYOND	II	NCT05852938
	BAFF and APRIL	Ataticept	ORIGIN-3	IIb	NCT04716231
	BAFF and APRIL	Telitaticept		III	NCT04905212
	BAFF and APRIL	Povetacicept		I/II	NCT05732402
Complement					
	MASP-2	Narsoplimab	ARTEMIS-IgAN	III	NCT03608033
	C Factor B	Iptacopan	APPLAUSE-IgAN	III	NCT04578834
	C5	Cemdisiran	ALN-CC5	II	NCT03841448
	C5	Ravulizumab	SANCTUARY	II	NCT04564339
Supportive care					
	Dual Endothelin and Angiotensin (DEARA)	Sparsentan	PROTECT	III	NCT03762850
	Endothelin A	Atrasentan	AFFINITY	II	NCT04573920
		Atrasentan	ALIGN	III	NCT04573478
	Endothelin A	SC 0062		II	NCT05687890

## Data Availability

Not applicable.
